# Characterization of Intestinal Bacteria in Wild and Domesticated Adult Black Tiger Shrimp (*Penaeus monodon*)

**DOI:** 10.1371/journal.pone.0091853

**Published:** 2014-03-11

**Authors:** Wanilada Rungrassamee, Amornpan Klanchui, Sawarot Maibunkaew, Sage Chaiyapechara, Pikul Jiravanichpaisal, Nitsara Karoonuthaisiri

**Affiliations:** 1 Microarray Laboratory, National Center for Genetic Engineering and Biotechnology (BIOTEC), Khlong Luang, Pathum Thani, Thailand; 2 Aquatic Molecular Genetics and Biotechnology Laboratory, National Center for Genetic Engineering and Biotechnology (BIOTEC), Khlong Luang, Pathum Thani, Thailand; The University of Plymouth, United Kingdom

## Abstract

The black tiger shrimp (*Penaeus monodon*) is a marine crustacean of economic importance in the world market. To ensure sustainability of the shrimp industry, production capacity and disease outbreak prevention must be improved. Understanding healthy microbial balance inside the shrimp intestine can provide an initial step toward better farming practice and probiotic applications. In this study, we employed a barcode pyrosequencing analysis of V3-4 regions of 16S rRNA genes to examine intestinal bacteria communities in wild-caught and domesticated *P. monodon* broodstock. Shrimp faeces were removed from intestines prior to further analysis in attempt to identify mucosal bacterial population. Five phyla, *Actinobacteria*, *Fusobacteria*, *Proteobacteria*, *Firmicutes* and *Bacteroidetes,* were found in all shrimp from both wild and domesticated environments. The operational taxonomic unit (OTU) was assigned at 97% sequence identity, and our pyrosequencing results identified 18 OTUs commonly found in both groups. Sequences of the shared OTUs were similar to bacteria in three phyla, namely *i) Proteobacteria* (*Vibrio*, *Photobacterium*, *Novosphingobium*, *Pseudomonas*, *Sphingomonas* and *Undibacterium*), *ii) Firmicutes* (*Fusibacter*), and *iii) Bacteroidetes* (*Cloacibacterium*). The shared bacterial members in *P. monodon* from two different habitats provide evidence that the internal environments within the host shrimp also exerts selective pressure on bacterial members. Intestinal bacterial profiles were compared using denaturing gradient gel electrophoresis (DGGE). The sequences from DGGE bands were similar to those of *Vibrio* and *Photobacterium* in all shrimp, consistent with pyrosequencing results. This work provides the first comprehensive report on bacterial populations in the intestine of adult black tiger shrimp and reveals some similar bacterial members between the intestine of wild-caught and domesticated shrimp.

## Introduction

Characterization of microbiota in animal intestines has been central in advancing understanding of the relationship between host and microorganism [1]–[3]. Microbial communities in humans and mice are estimated to comprise >1,000 taxa [1], [3]. While some microbes can be pathogenic to their hosts, other microbial symbionts are beneficial to the development and physiology of their host [4]–[6], playing roles in nutrient absorption, immune response, and epithelial development [7]–[9]. Maintaining a balance in the population of intestinal bacteria is crucial to the health of the host [10]. In turn, several host factors, such as diet, developmental stage and physiological condition have been identified to affect intestinal bacterial composition [1], [11], [12]. The host, by its own gut immune system and gut environment (e.g. pH and bile acids), also shapes the composition of intestinal bacteria to maintain a relatively stable level of diversity [13], [14].

In addition to understanding the host-microbiota relationship, characterization of intestinal bacteria will help identify bacteria with potential to become probiotics, which are increasingly used as an alternative means for preventive medicine in humans and animals [15]–[18]. In aquatic animal farming, the concept of replacing antibiotic use with probiotics will help prevent environmental contamination and adverse health effects for consumers. Indeed, several studies have developed probiotic applications for aquaculture [19]–[21]. This is no exception for *Penaeus monodon* (black tiger shrimp), which has been a commercially important marine crustacean for the past few decades in many Asian countries and Australia [22], [23]. With increasingly high shrimp consumption, the decline of wild harvests has forced domestication to become the major source of shrimp production [24]. However, the farming industry has been deteriorating due to several factors, in particular the outbreak of disease.

As alternative means to address the disease outbreak problem, disease control using probiotics or maintaining healthy intestinal bacterial balance requires comprehensive understanding of bacterial diversity in the intestines of commonly farmed aquatic animals. Although there have been several studies on bacterial diversity in commonly cultured aquatic species animals from the wild such as *P. merguiensis* (banana shrimp) [25], *Salmo salar* L (Atlantic salmon) [26], *Gadus morhua* L (Atlantic cod) [27], and *Danio rerio* (zebrafish) [2], there are limited findings on the intestinal bacteria of *P. monodon.* It was only recently that studies of bacterial diversities in the intestines of *P. monodon* post-larval and juvenile stages were reported [28], [29], yet there has been no report on the intestinal bacteria of wild-caught *P. monodon*, which feed on slow-moving benthic animals such as small crabs, shrimp, mollusks, and polychaetes [30]. In contrast, domesticated shrimp in rearing facilities are fed with commercial feed pellets and reared in monoculture with higher stocking density. Comparing the differences in intestinal bacteria of wild-caught and domesticated shrimp can unveil changes in intestinal bacteria as shaped by aquaculture. These changes have implications for shrimp health and consequently, application of this knowledge may improve survival rates of *P. monodon* under domestication.

In this study, we examined bacterial populations associated with the intestines of wild-caught and domesticated adult black tiger shrimp using high-throughput next-generation pyrosequencing analysis in parallel with denaturing gradient gel electrophoresis (DGGE). Similarities and differences in intestinal bacteria between wild and domesticated shrimp provide initial evidence for a core bacterial community as well as probiotic candidates for *P. monodon*.

## Materials and Methods

### Ethics Statement

An ethics statement is not required for this work. No specific permits were required for the described field studies. The field location is not privately owned or protected in any way, and the field studies did not involve endangered or protected species.

### Intestine Sample Collection from Wild-caught and Domesticated Black Tiger Shrimp Broodstocks

**Figure 1:**
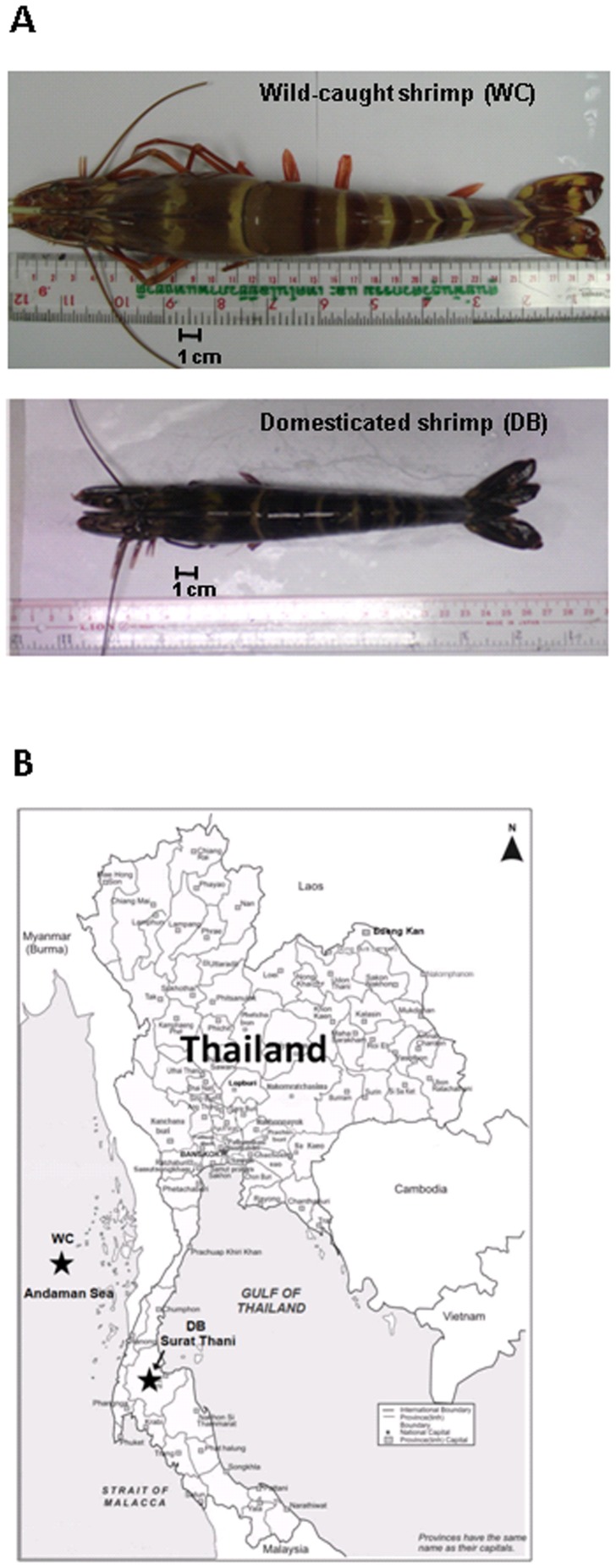
*Penaeus monodon* from wild and domesticated habitats (A). Wild-caught *P. monodon* adults (WC) were captured from the Andaman Sea, whereas 10-month-old domesticated adults (DB) were reared at the Shrimp Genetic Improvement Center, Surat Thani, Thailand (B). The map was adapted from http://www.mapsofworld.com/thailand/thailand-political-map.html.

Wild-caught broodstocks (WC, n = 3) were obtained from the Andaman Sea (salinity ∼31 ppm) and 10-month-old domesticated broodstocks (DB, n = 3) were reared in a cement tank with seawater pumping in from the Gulf of Thailand (salinity ∼30 ppm) at the Shrimp Genetic Improvement Center (Surat Thani province, Thailand) (Fig. 1). The domesticated shrimp were fed three times per day with commercial pellets containing >35% protein, 5%> lipid, <12% moisture and <4% ash (Charoen Pokphand Food PCL, Thailand). Average shrimp weight was 179.03±14.89 g for WC, and 44.94±7.08 g for DB. Shrimp intestines from each group were aseptically dissected, and fecal matter was removed before storage in liquid nitrogen. Genomic DNA was extracted from individual intestines using the QIAamp DNA stool mini kit (Qiagen).

### Barcode Pyrosequencing Analysis for Bacterial Diversity in Shrimp Intestines

The primer pair 338F (5′ ACTCCTACGGGAGGCAGCAG 3′) and 786R (5′ CTACCAGGGTATCTAATC 3′), targeting the V3 and V4 variable regions of the 16S rRNA gene (between nucleotide positions 338 and 786) was modified with a barcode tag by adding a random 8-base sequence to the 5′ end of each primer pair. DNA libraries for bacterial diversities in WC and DB intestines were amplified with the barcode-tagged primer pairs 338F and 786R using high-fidelity DNA polymerase (KOD hotstart, Novagen, USA). The PCR products were purified with Qiaquick gel extraction kit (Qiagen), and then ligated to 454-adapters. The samples were sent for pyrosequencing at the Genomic Research Laboratory, Genome Institute, BIOTEC, using the Genome Sequencer platform according to Roche protocol (454 GS FLX System, Roche, USA) [31], [32].

### Pyrosequencing Data Analysis

Pyrosequencinc data were processed using Mothur software to remove low quality sequences [33], which were defined as sequences with <200 bases, with ambiguous bases and homopolymers >6 bases, or without a barcode and a primer sequence. Sequences identified as chimeras by UCHIME [34] were removed before the RDP II Classifier analysis using an 80% confidence threshold [35]. Mothur was used to assign operational taxonomy units (OTUs) using the average neighbor algorithm at 97% similarity level [36], and to calculate Shannon and Chao1 indices for each shrimp library. In addition, the coverage index was calculated by 1−(n/N), where n is the number of phylotypes and N is the total number of sequences [37]. A hierarchical tree was constructed for each library to visualize relative abundance of each genus from wild and domesticated shrimp. MEGAN was used to construct taxonomic trees using taxonomical assignment by the RDP classifier [38]. Principal coordinates analysis (PCoA) was assessed with R script to compare bacterial community structures based on weighted-UniFrac from each library. Statistical analyses of weighted-UniFrac distances were calculated by analysis of molecular variance (AMOVA) in Mothur to compare significant differences between bacterial communities in wild and domesticated shrimp [39], [40]. A calculated *P*-value <0.05 was considered to be statistically significant. All pyrosequences in this study were deposited into GenBank database with accession numbers: KF329429–KF334451 for the WC1 library, KF334452–KF344403 for the WC2 library, KF344404–KF355928 for the WC3 library, KF322280–KF325238 for the DB1 library, KF325239–KF328420 for the DB2 library, and KF328421–KF329428 for the DB3 library.

### PCR-denaturing Gradient Gel Electrophoresis Analysis and Statistical Analysis of Bacterial Profiles

Denaturing gradient gel electrophoresis (DGGE) was performed to analyze bacterial profiles of individual shrimp. The 16S rRNA PCR fragments were amplified with genomic DNA from each intestine (50 ng) as the template with primer pair, 338F-GC (5′ CGCCCGCCGCGCGCGGCGGGCGGGGCGGGGGCACGGGGGG ACTCCTACGGGAGGCA 3′) and 518R (5′ ATTACCGCGGCTGCTGG 3′), in which the underlined sequence represents the GC-clamp region [41]. PCR cycling conditions involved initial denaturation at 94°C for 3 min, 30 cycles of denaturation at 94°C for 30 sec, annealing at 55°C for 1 min, and extension at 72°C for 2 min, and final extension at 72°C for 5 min. PCR products were purified by gel extraction (Illustra GFX PCR purification kit, GE Healthcare), and determined concentration by a spectrophotometer (NanoDrop ND8100). DGGE was performed as previously described [29] with slight modifications. Briefly, 600 ng of PCR-DGGE fragments were loaded into each well of an 8% polyacrylamide gel with a 40–60% denaturing vertical gradient. A DNA standard of known PCR-DGGE fragments prepared in-house was used to ensure efficiency and quality of DGGE separation. Electrophoresis was performed at 80 volts for 14 hr in DCODE universal mutation detection system (BIO-RAD, USA) before the gels were stained in SYBR gold (Invitrogen) according to the supplier’s instructions. Selected DNA bands from DGGE were excised, transferred to 50 µl of sterile TE buffer, and incubated overnight at 4°C. DNA in TE (1 µl) was used as the template for PCR amplification with a primer pair containing M13 priming sequences (underlined) (338GCF-M13R 5′ CAGGAAACAGCTATGA CGGGCGGGGCGGGGGCACGGGGGGACTCCTACGGGAGGCA 3′ and 518R-AT-M13F 5′ GTAAAACGACGGCCAG TAAATAAAATAAAAATGTAAAAAAATTACCGCGGCTGCTGG 3′) [42]. The purified PCR product of each DGGE band was directly sequenced using the M13R primer. The obtained sequences were compared to the ribosomal database (http://rdp.cme.msu.edu/) to identify sequences of nearest similarity, where a sequence with similarity greater than 97% was classified to the closest type species [35], [43], and similarity of 90–97% was classified to the nearest genus. DGGE profiles were clustered using the unweighted-pair-group method with arithmetic averages (UPGMA) based on Pearson correlation of densitometric curves by InfoQuestFP (Biorad, USA).

## Results

### Overview of 16S rRNA Pyrosequencing Analysis

Barcode pyrosequencing of 16S rRNA sequences was employed to determine bacterial populations in intestines of six individual adult shrimp from wild (WC, n = 3) and domesticated (DB, n = 3) habitats (Fig. 1). A total of 61,499 reads were obtained from pyrosequencing of the V3-4 regions of 16S rRNA genes (Table 1). The barcodes assigned the sequences to distinct libraries: WC1 (5,029 sequences), WC2 (9,969 reads), WC3 (11,532 reads), DB1 (2,961 reads), DB2 (3,190 reads) and DB3 (1,009 reads). Sequences were clustered into operational taxonomic units (OTUs) at 0.03 dissimilarity levels, in which each OTU represented a unique phylotype. The total numbers of OTUs ranged from 138 to 806, where the WC group exhibited higher variation in the number of OTUs. The OTUs were further classified to genus level using the RDP II Classifier [35]. All six bacterial communities contained five phyla: *Actinobacteria, Bacteroidetes, Firmicutes, Fusobacteria,* and *Proteobacteria*. DB1 from the DB group had the lowest number of bacteria genera (21, 43 and 28 genera for DB1, DB2 and DB3, respectively), whereas the highest number of bacteria genera was detected in WC2 from the WC group (39, 89, and 52 genera for WC1, WC2 and WC3, respectively).

**Table 1:** Summary of pyrosequencing read analysis, bacterial diversity richness (OTUs), sample coverage (Good’s coverage), diversity index (Shannon) and estimated OTU richness (Chao1) for intestinal bacterial diversity analysis of wild-caught (WC, n = 3) and domesticated (DB, n = 3) *P. monodon*.

	WC			DB		
	1	2	3	1	2	3
**Sampling depth**						
A total number of sequences	5,023	9,952	11,525	2,959	3,182	1,008
OTUs (97%)	138	566	806	254	395	138
Phylum	5	5	5	5	5	5
Class	8	10	13	9	11	10
Family	28	48	38	21	35	21
Genus	39	89	52	21	43	28
**Diversity indices**						
Good’s Coverage	0.95	0.97	0.95	0.94	0.91	0.90
Shannon	2.45	2.66	3.21	2.61	3.58	2.73
Chao1	1,003	1,663	2,454	855	1,413	597

**Figure 2:**
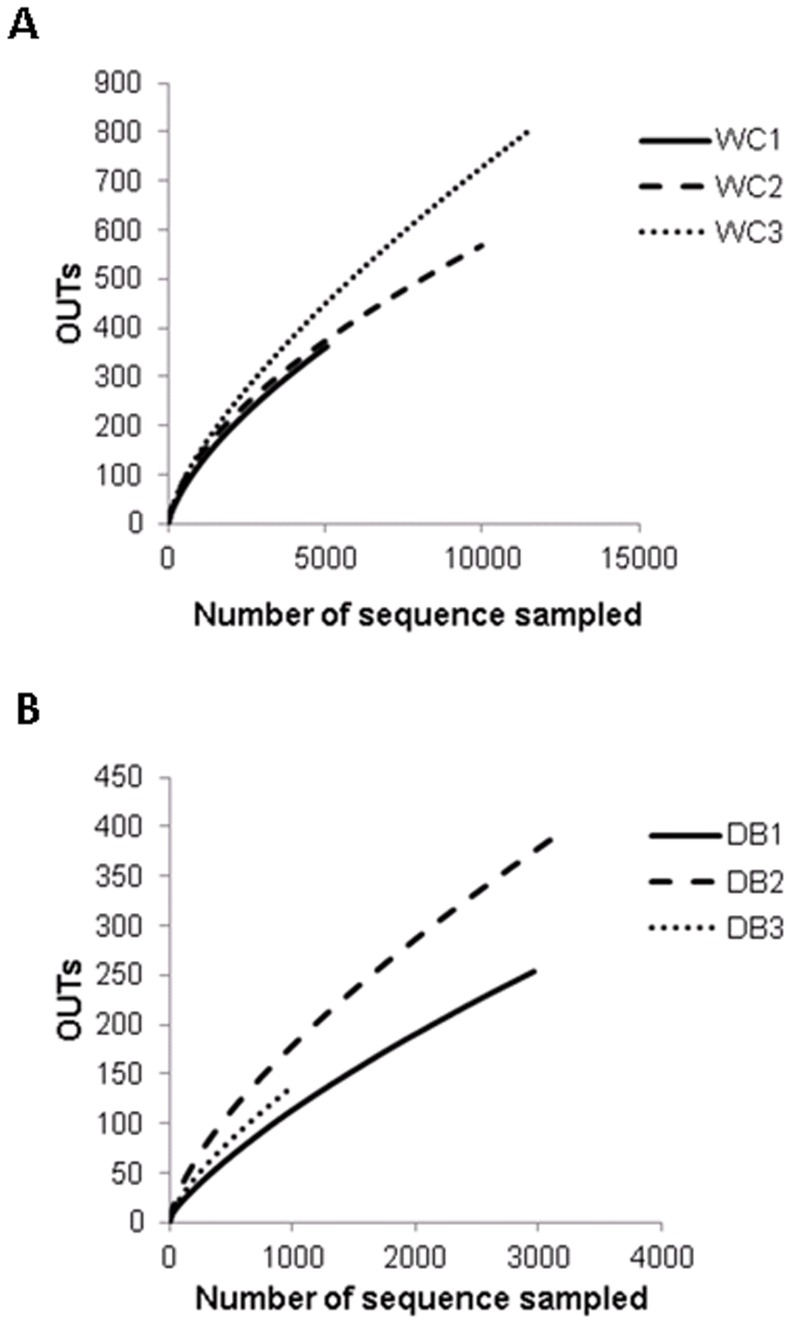
Rarefaction analysis of shrimp intestines. Operational taxonomic units (OTUs) were clustered based on 97% sequence similarity for the (A) wild-caught group (WC1, WC2, and WC3) and (B) the domesticated group (DB1, DB2, and DB3). The number of sequences sampled represents the number of pyrosequencing reads.

To estimate and compare bacterial diversity in each individual shrimp, bacterial diversity indices were calculated from OTUs of each library. Good’s coverage indices, used to estimate the percentage of total bacterial OTUs represented in a sample, were in the range of 0.90–0.97, suggesting the 16S rRNA results from each library represented the majority of bacteria in the shrimp intestines. Bacterial diversity estimated by the Shannon index varied from 2.45 to 3.21 in the WC group, and 2.61–3.58 in the DB group, suggesting a similar range of diversity between the two environments. Chao1 analysis for bacterial richness estimated a range of 597 to 2,454 phylotypes, higher than the actual observed OTUs in each library, indicating that the true bacterial richness in *P. monodon* intestines was underestimated. Additionally, rarefraction analysis was carried out to determine whether OTUs from each library had been adequately obtained from pyrosequencing analysis (Fig. 2). Consistent with the Chao1 analysis, rarefraction curves for the WC (Fig. 2A) and DB groups (Fig. 2B) did not plateau, suggesting that bacterial richness of adult *P. monodon* intestines were not yet determined. Additional sequence sampling will still be required to capture the true intestinal bacterial diversity of adult *P. monodon*. Even with the help from the next-generation pyrosequencing technique, a microbial ecology study to reveal a complete picture of bacterial communities is still very challenging. For instance, in soil bacteria community analyses, rarefraction curves have not been saturated, even when a range of 5,000 to 10,000 pyrosequencing reads were obtained [44].

### Taxonomic Composition of Intestinal Bacteria in P. monodon from Pyrosequencing Data

**Figure 3:**
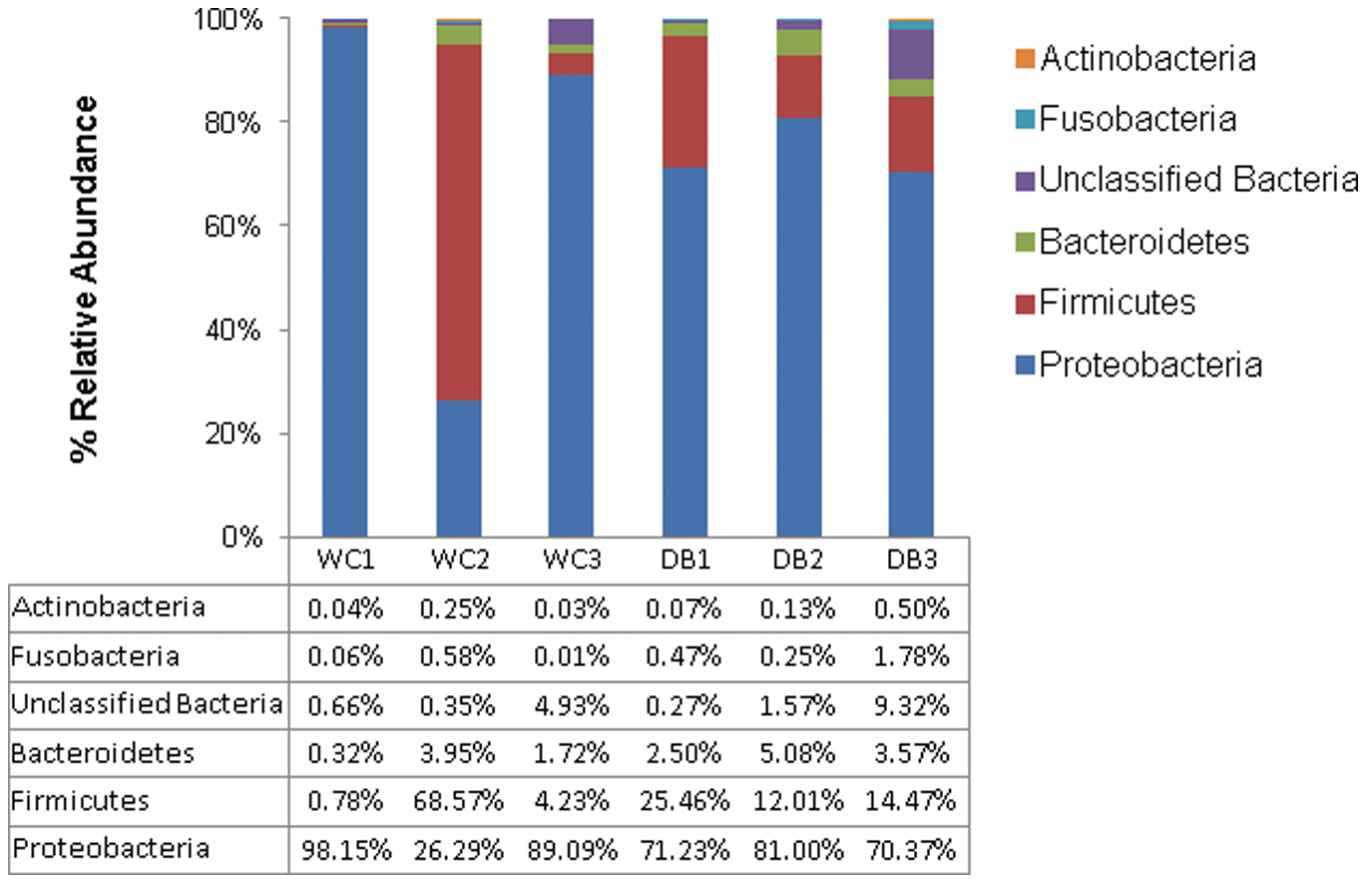
Frequency distribution of bacterial phyla in *Penaeus monodon* intestines from pyrosequencing libraries of the wild-caught group (WC1, WC2 and WC3), and the domesticated group (DB1, DB2 and DB3).

**Figure 4:**
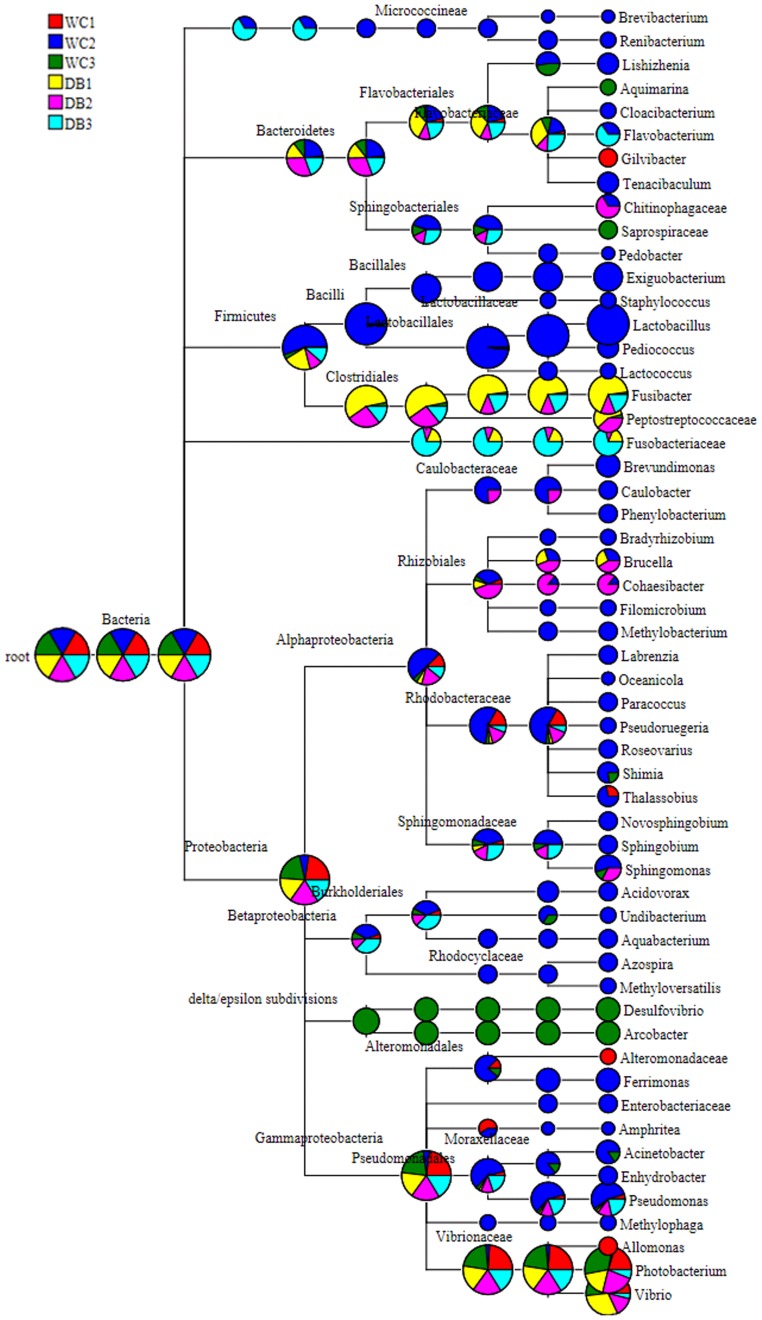
Comparison of bacterial compositions in wild-caught (WC1, WC2 and WC3) and domesticated (DB1, DB2 and DB3) shrimp intestines. Pie charts of the hierarchical tree reflect relative abundance for each genus from each library (red represents DB1, blue represents DB2, green represents DB3, yellow represents WC1, pink represents WC2, and light blue represents WC3).

The five major phyla associated with *P. monodon* intestines were *Actinobacteria*, *Fusobacteria*, *Bacteroides*, *Firmicutes* and *Proteobacteria* (Fig. 3). Among them, *Proteobacteria* (>70% of total sequences) dominated in all shrimp intestines, followed by *Firmicutes*, except for the WC2 library. WC2 library had 69% of total sequences belonging to *Firmicutes*, and 26% of total sequences were from *Proteobacteria*. Considering intra-group diversity, WC shrimp had more variation in phyla distribution whereas DB shrimp had relatively similar distributions, with *Proteobacteria* being the major phylum followed by *Firmicutes* (Fig. 3). To visualize bacterial composition in hierarchical orders, pie charts were constructed to represent bacterial abundance in intestines of WC and DB *P. monodon* (Fig. 4 and Fig. S1). Only phyla containing >100 total pyrosequencing reads were constructed into taxonomic nodes. *Vibrio* and *Photobacterium* were dominant in all six libraries, suggesting that these bacterial genera were well-adapted to conditions in *P. monodon* intestines and surrounding aquatic environments. Surprisingly, *Lactobacillus* was also present at high abundance in the WC2 library (Fig. S1A). *Lactobacillus* can be found in intestines of aquatic animals and marine environments [45], [46], thus, the unexpectedly high abundance of *Lactobacillus* in the WC2 library could be possibly acquired from ingested food.

While the WC libraries were widely distributed in all taxonomic nodes, the DB libraries were mostly distributed in classes *Gammaproteobacteria* and *Clostridia* (Fig. S1). Interestingly, *Fusibacter* (class *Clostridia*) were found in higher proportions in the DB group than the WC group (Fig. S1B). Bacterial frequency and their classification are summarized in Table S1. Pyrosequencing reads that could not be classified at genus level were classified to the nearest taxonomic ranking (Table S1).

### Evidence for Common Intestinal Bacterial Populations in P. monodon Adults

**Figure 5:**
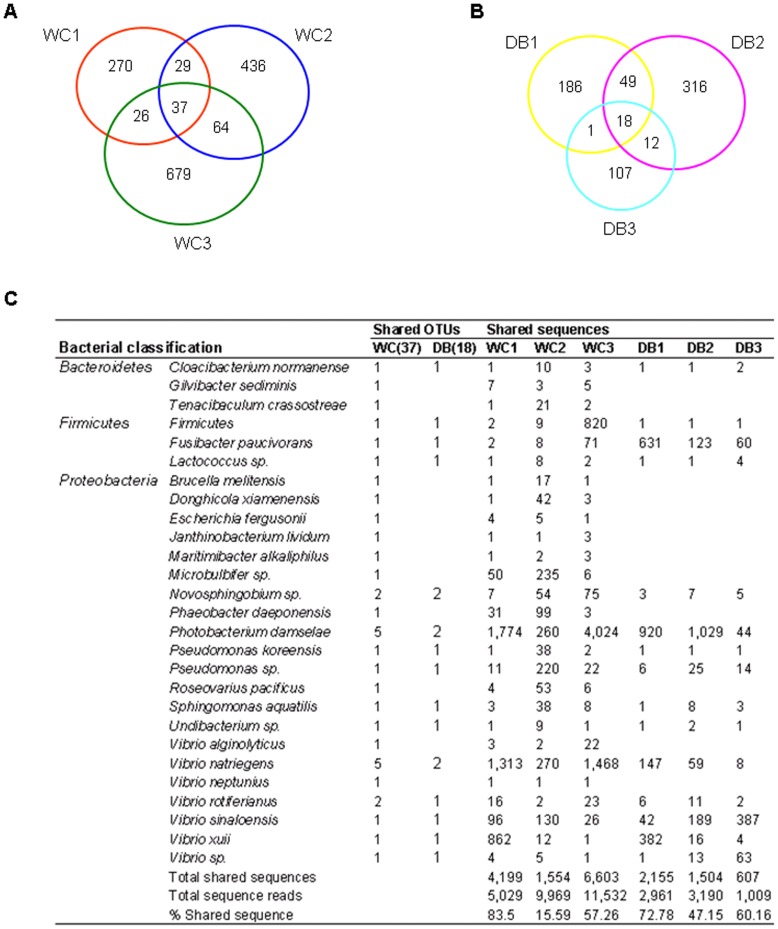
Shared OTU analysis of pyrosequencing reads from *Penaeus monodon* intestines. The Venn diagram represents the unique and shared OTUs at 97% similarity level in libraries of (A) wild-caught group (WC1, WC2, and WC3) and (B) the domesticated group (DB1, DB2 and DB3). The shared reads were classified based on BLAST (C) with the following criteria; similarity of ≥97% were classified to the nearest type strain, ≥90% to the nearest genus, and ≤90% to the nearest phylum.

From the OTU (97% similarity) distribution among the six libraries (Fig. 5), 1,541 OTUs were found to be unique within the WC libraries, and only 37 OTUs were shared within the group (2.4%) (Fig. 5A). On the other hand, 689 unique OTUs were obtained within the domesticated libraries, and 18 OTUs were common within this group (2.6%) (Fig. 5B), reflecting bacterial variations among individual WC and DB *P. monodon.* These bacteria could perhaps represent transient bacterial populations. Shared OTUs were assigned to the nearest species based on Blast analysis to determine common bacterial species (Fig. 5C). Interestingly, all 18 OTUs shared in the domesticated libraries were a subset of those 37 OTUs shared within the WC libraries, and these may be part of the core intestinal bacteria in *P. monodon* adults. The shared OTUs were classified to *Proteobacteria*, *Firmicutes* and *Bacteroidetes*. Phylum *Proteobacteria* contained the most shared OTUs among the six libraries, comprising *Novosphingobium*, *Photobacterium*, *Pseudomonas*, *Sphingomonas*, *Undibacterium*, and *Vibrio* (Fig. 5C). Among *Firmicutes*, a shared OTU was classified to *Fusibacter paucivorans*, whereas the other OTU could only be classified to phylum level. Only *Cloacibacterium normanense* from phylum *Bacteroidetes* was common across the WC and DB libraries.

On the other hand, there were distinct differences between the microbial structures of the WC and the DB shrimp. From 37 OTUs found from the WC libraries, the 19 OTUs absent in the domesticated included several bacteria previously found in marine sediment or marine organisms such as *Gilvibacter sediminis* [47], *Microbulbifer* sp. [48], *Donghicola xiamenensis* [49],*Tenacibaculum crassostreae* [50], *Maritimibacter alkaliphilus* [51], *Phaeobacter daeponensis* [52], and *Roseovarius pacificus* [53]. In addition, two *Vibrio* species were found only in the WC. Interestingly, an antibiotic-producing bacterium *Janthinobacterium lividum* was also found only in the WC [54].

**Figure 6:**
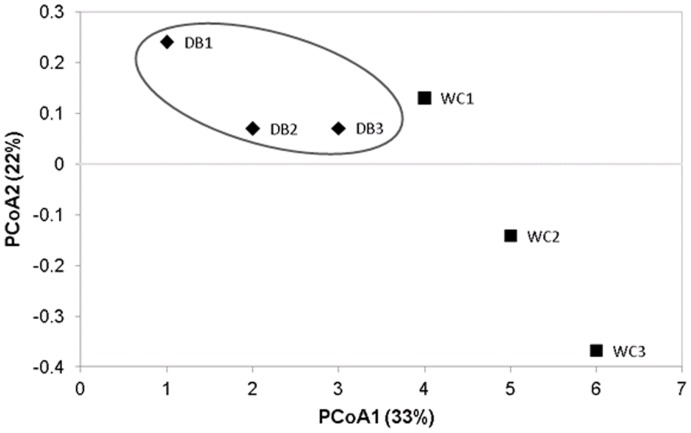
Principal coordinate analysis (PCoA) based on weighted-UniFrac analysis of bacterial populations associated with *P. monodon* intestines from the wild-caught group (WC1, WC2, and WC3) and the domesticated group (DB1, DB2 and DB3).

Principal coordinate analysis (PCoA) based on weighted-UniFrac distance showed that bacterial communities in the intestines of each shrimp in the WC were separated from one another, suggesting individual variation, whereas the DB group were clustered closer together (Fig. 6). The PCoA pattern suggested that bacterial populations in DB *P. monodon* showed more similarities within the group whereas the WC shrimp had higher variation. However, the statistical analysis of intestinal bacterial diversity did not show significant differences (*P*-value >0.05) between the WC and DB shrimp.

### Analysis of Bacterial Diversity by Denaturing Gradient Gel Electrophoresis

**Figure 7:**
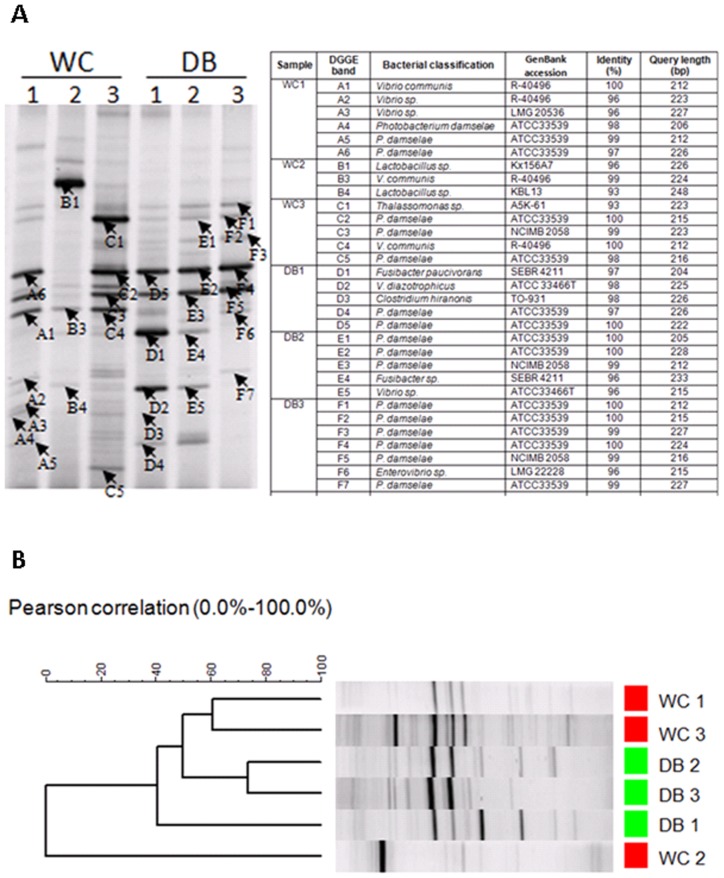
Denaturing gradient gel electrophoresis (DGGE) analysis of intestinal bacterial communities in *Penaeus monodon*. The bacteria profiles were from intestines of wild-caught shrimp (WC1, WC2, and WC3) and domesticated shrimp (DB1, DB2, and DB3). The selected DGGE bands were sequence to identify bacteria (A) and DGGE profiles were clustered using UPMGA based on Pearson correlation (B).

The bacterial patterns in intestines of WC and DB shrimp were compared by denaturing gradient gel electrophoresis (DGGE) (Fig. 7). DGGE bands from each sample were selected for sequencing (Fig. 7A). The majority of DGGE bands from all six libraries were highly similar to bacteria from *Vibrio* and *Photobacterium.* One dominant DGGE band similar to *Lactobacillus* sp. (Fig. 7A, band B1) was only found in WC2. A sequence similar to *Thalassomonas* sp. (band C1) was also found in WC3. Sequences of DGGE bands similar to *Fusibacter* were found in DB1 and DB2. Consistent with the pyrosequencing result, cluster analysis of DGGE band profiles based on the Pearson distance matrix revealed that bacterial populations in WC shrimp showed higher individual variation than those in DB shrimp (Fig. 7B).

## Discussion

The influence of intestinal bacteria on their hosts has been mostly elucidated in vertebrate hosts, with profound effects on host genes involved in nutrient absorption, mucosal modification and immune response. Some intestinal bacteria have been linked to the health status of their hosts [16]. Due to the influence of intestinal microbiota on animal fitness, characterization of gut microbiota would be essential for farm animals. Here, we characterized bacterial populations associated with the intestines of *P. monodon*, an economically important shrimp species in Asia [55]. To identify a core bacterial population, intestinal bacterial communities were determined in *P. monodon* obtained from wild and domesticated environments using a high-throughput pyrosequencing approach in parallel with DGGE analysis.

In this study, pyrosequencing analysis revealed some OTUs shared among *P. monodon* intestines from wild and domesticated environments. Although these OTUs represent only a small percentage of the total intestinal bacterial population, the dominant OTUs in each shrimp were commonly found in all six shrimp libraries. The closest relatives were classified to six genera from *Proteobacteria* (*Vibrio*, *Photobacterium*, *Pseudomonas*, *Sphingomonas*, *Novosphingobiumcommon* and *Undibacterium*), two genera from *Firmicutes* (*Fusibacter* and *Lactobacillus*), and one genus from *Bacteroidetes* (*Cloacibacterium*). Among the shared bacterial genera in this study, *Vibrio*, *Photobacterium*, *Pseudomonas*, *Sphingomonas* and *Novosphingobium* were found to be highly prevalent in the intestines collected from *P. monodon* post-larva and juveniles in our previous study [29]. In particular, *Vibrio* and *Photobacterium* are commonly associated with marine habitats [56] and many marine organisms such as crustaceans [25], [57], oyster [58], and fish [59]. Although the bacteria in these genera have been characterized as free-living or commensal in marine animal digestive tracts, the consistent detection of *Vibrio*, *Photobacterium*, *Pseudomonas*, *Sphingomonas* and *Novosphingobium* genera in this work and previous studies could imply that they are an indigenous bacterial population in *P. monodon* intestines. Some have been reported as pathogens in aquaculture, such as *Vibrio harveyi,* an opportunistic pathogen that causes infection in shrimp under conditions of high nutrient concentrations and high animal density in rearing environments [60], [61]. *Fusibacter* were commonly found in shrimp from both groups. Thus far, bacteria isolates belonging to the genus *Fusibacter* have been found to be strictly anaerobic and halotolerant bacteria [62], [63]. Interestingly, *Lactobacillus* was also found to be associated with *P. monodon* intestines. Members of *Lactobacillus* are indeed employed as probiotics in many animals, including some aquatic organisms [64], [65]. For instance, the group of *Litopenaeus vannamei* (Pacific white shrimp) fed with *Lactobacillus plantarum* supplemented diet shows a higher survival rate under pathogen exposure than the control diet group [66]. The detection of *Lactobacillus* in *P. monodon* intestines in this report as well as our previous work [29] also suggests that the bacterium could withstand *P. monodon* gut and aquatic environments. Hence, application of *Lactobacillus* as probiotics is a promising mean to enhance disease resistance in the *P. monodon* farming. Despite diverse intestinal bacteria members associated in *P. monodon* adults, the dominant bacteria detected in intestines of WC and DB *P. monodon* were *Proteobacteria,* followed by *Firmicutes* and *Bacteroidetes*. This observation is consistent with previous reports on intestinal bacteria in *P. monodon* post-larvae and juveniles [28], [29]. The prevalence of these phyla in animal intestines has been reported in aquatic organisms such as *Fenneropenaeus chinensis* (Chinese shrimp), *P. merguiensis* (banana prawn), *Nephrops norvegicus* (Norway lobster) and *Danio rerio* (zebrafish) [2], [25], [67], [68].

The similarity of bacterial communities among the wild and domesticated shrimp suggested the establishment of specific bacteria by selective pressures from the environment within the host gut [69], [70]. For instance, a germ-free mice model study reveals that mucosal environment influences selection and establishment of bacteria in the gastrointestinal tract [3]. The mucosal immune system in a host’s digestive tract has been demonstrated to play important roles on maintaining commensal bacteria [69], [71]. Although understanding of *P. monodon* gut physiology remains elusive, expression of *P. monodon* immune-related genes upon pathogenic bacteria challenge provides evidence of a shrimp gut immune system [72]. Furthermore, the observation of shared bacterial members is congruent with previous comparative studies of other wild and domesticated model animals. For instance, comparison of bacteria in intestines of *D. Rerio* (zebrafish) from wild and lab-reared environments showed similar bacterial members [2]. Analyses of bacterial communities in wild and domesticated *Mus musculus* (mice) [73], *Drosophila melanogaster* (fruit flies) [74] or *Hydra* species (Hydra) [70] reveal shared bacterial members regardless of individual variation, and similar bacteria compositions were observed at the phylum or class level in both wild and domesticated hosts. Finally, the similarities of intestinal bacteria in *P. monodon* with different life histories also imply that there has been long-term co-evolution between intestinal bacteria and host shrimp.

Although shared OTUs were observed among intestines of the six adult shrimps, there were still a vast number of unique OTUs, which reflect the high degree of bacterial variation among individual *P. monodon*, especially in wild-caught shrimp. In this study, bacterial profiles from the DGGE analysis were congruent with pyrosequencing results in that there was higher individual variation in intestinal bacterial populations among the wild-caught than the domesticated shrimp. As intestinal bacteria can be influenced by bacteria present in the surrounding aquatic habitats [75]. Domesticated shrimp reared in the same water tank would therefore experience less fluctuation in bacterial composition in the surrounding water compared to wild habitats. Transiently persistent bacteria and those associated with ingested food could also inflate the observed individual bacterial variation. As wild *P. monodon* fed on smaller live animals, unlike domesticated shrimp fed mainly with dried feed pellets, the small live animals ingested by wild *P. monodon* might influence the number of intestinal bacteria genera, resulting in higher numbers of genera detected in the wild-caught *P. monodon* than the domesticated group. To further determine the effect of diets on intestinal bacteria, the characterization of bacterial compositions in *P. monodon* intestines is underway to compare differences between *P. monodon* feeding on live food (e.g. polychaete worms) to those fed with commercial feed pellets.

Most intestinal bacterial population analyses in penaeid shrimp have been characterized by both culture-dependent and culture-independent techniques based on Sanger sequencing of 16S rRNA genes [25], [28], [76] and a recent study has applied the high-throughput 454 pyrosequencing platform to the analysis of intestinal bacterial diversity in *P. monodon* [29]. The introduction of pyrosequencing technology has enabled a greater depth to bacterial population analysis in various environments; for instance, bacterial richness in the deep sea environment has been reported to be one or two orders of magnitude higher than those obtained under the traditional Sanger based approaches [77]. Despite its advantages, even pyrosequencing may not uncover the entire range of bacterial diversity. Although the reading length obtained from pyrosequencing technology has been continuously improving, the full length of 16S rRNA (∼1.5 kb) has not yet been achieved with current 454 pyrosequencing capability [78]. Primer selection and their target regions influence bacterial diversity analyses [79], [80], and indeed the detection of phyla *Verrucomicrobia*, *Planctomycetes* and *Chlamydiae* is highly variable with 16S rRNA primer sets [79]. We chose primers target variable regions 3 and 4 of the 16S rRNA gene, which may not be effective in detecting the presence *Verrucomicrobia* and *Planctomycetes* [80]. Nonetheless, these bacteria phyla were not found in high abundance in *P. monodon* intestines in a previous study using full-length 16S rRNA clone library approach [28]. While pyrosequencing analysis provided more comprehensive view of bacterial diversity in a community, the DGGE analysis in parallel provided an overview of structures of dominant bacterial groups, which can be used for comparison of bacterial patterns [81].

Our work reports intestinal bacterial composition in wild-caught and domesticated *P. monodon* adults by using the 454-pyrosequencing approach and bacterial patterns were also compared by DGGE analysis. We provided evidence of common intestinal bacteria in *P. monodon* intestines, and suggest that the intestine may be viewed as a selective environment that allows only certain bacterial taxa to persist. Characterization of the bacterial composition in *P. monodon* intestines is fundamental to understanding of the beneficial effects of bacteria and the importance of host-microbe interaction in a non-model organism.

### Conclusion

Recent studies have reported the importance of balanced gut microbiota to the health of animal hosts. Hence, the main purposes of this study were to utilize a culture-independent high-throughput pyrosequencing technique to extend our knowledge concerning the breadth of bacterial diversity in intestines of adult *P. monodon*, and to determine the composition of bacterial communities in *P. monodon* from the wild and those under domestication. The bacterial profiles showed similar dominant genera in wild-caught and domesticated shrimp, suggesting the occurrence of a resident bacterial population in *P. monodon*. We identified shared bacterial members associated with adult shrimp intestines from wild and domesticated environments. This work provides evidence that host intestinal conditions exert stronger selective pressure for bacterial community establishment in *P. monodon* than rearing environments.

## Supporting Information

Figure S1 **The hierarchical trees reflect bacterial abundance for (A) wild-caught shrimp (WC1, WC2, and WC3) and (B) domesticated shrimp (DB1, DB2, and DB3).**
(DOC)

Table S1 **Taxonomical assignments of 16S rRNA sequences from pyrosequencing using the RDP classifier with a confidence threshold of 80%.**
(DOC)
