# Supplementary figures and images for: Characterization of Intestinal Bacteria in Wild and Domesticated Adult Black Tiger Shrimp (Penaeus monodon)
Source: PLoS One. 2014 Mar 11;9(3):e91853. doi: 10.1371/journal.pone.0091853 (PMC3950284; doi:10.1371/journal.pone.0091853)

**Fig. S1**

**A**

**
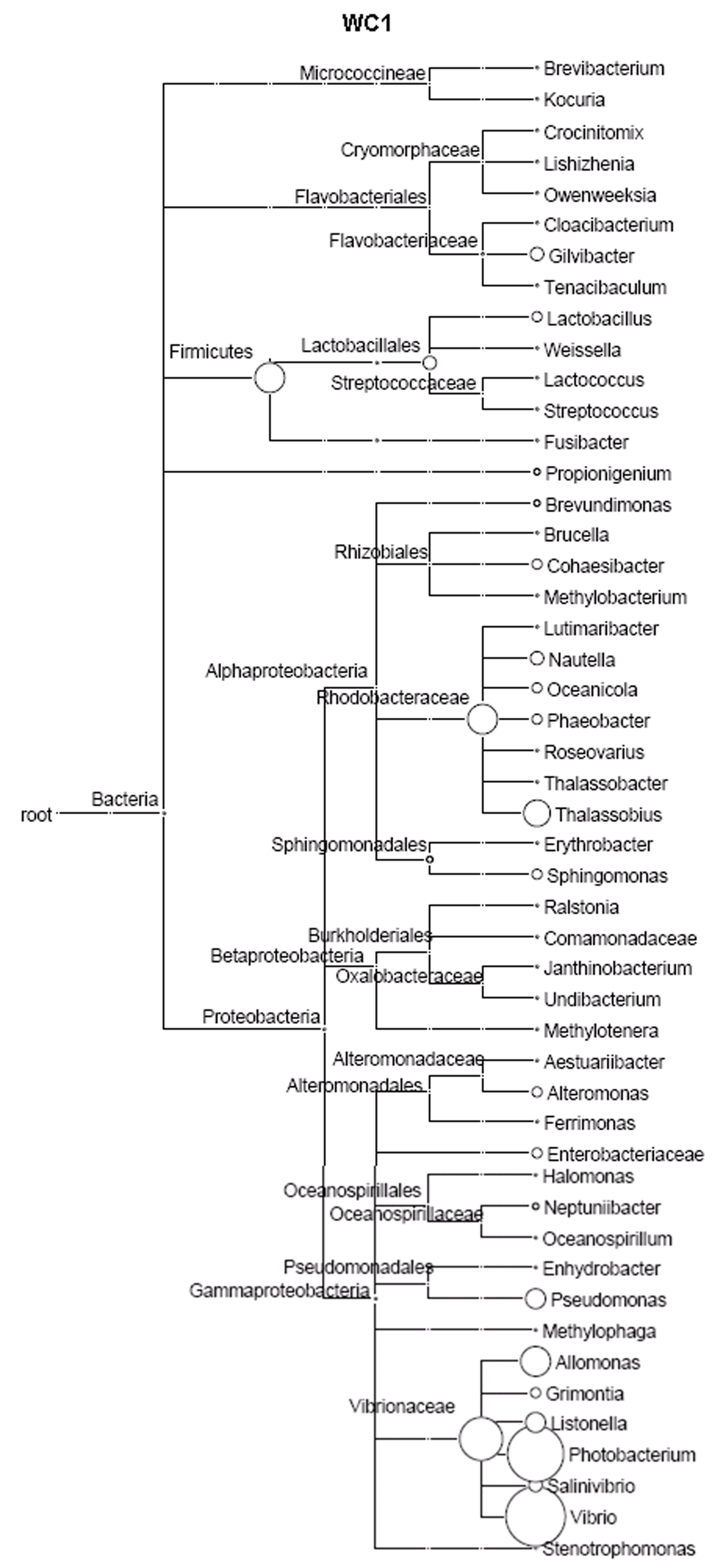

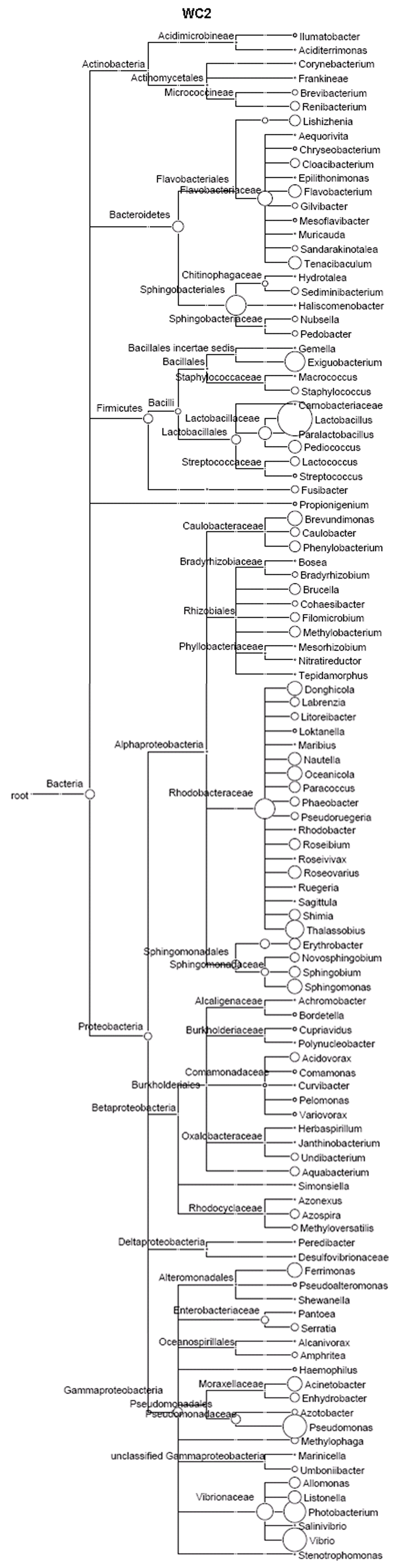

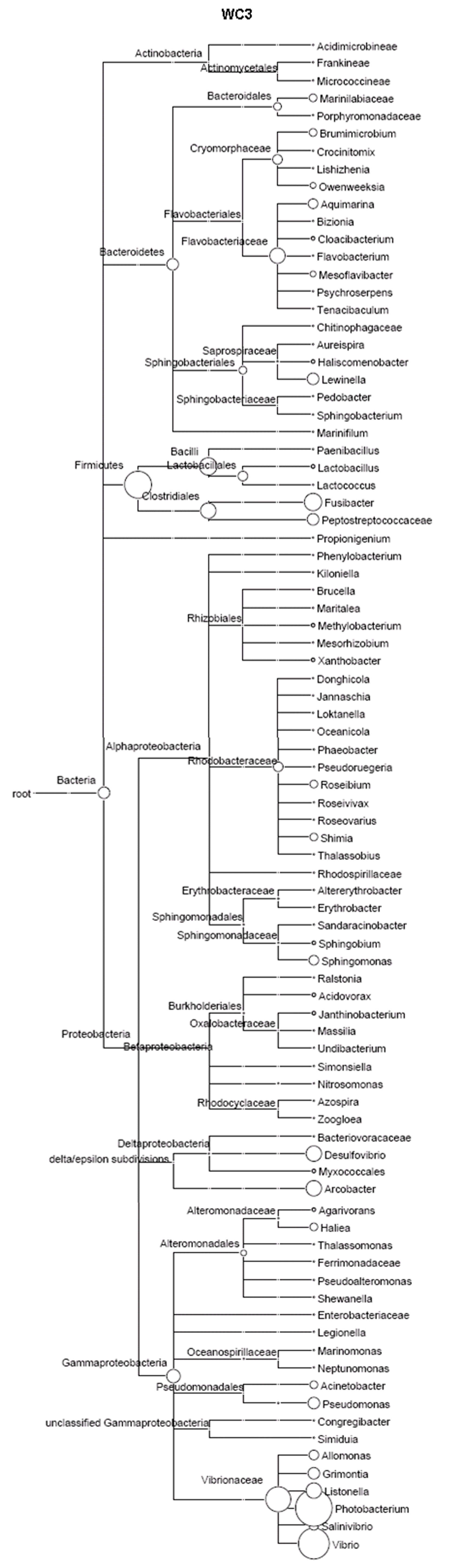
**

**B**

**
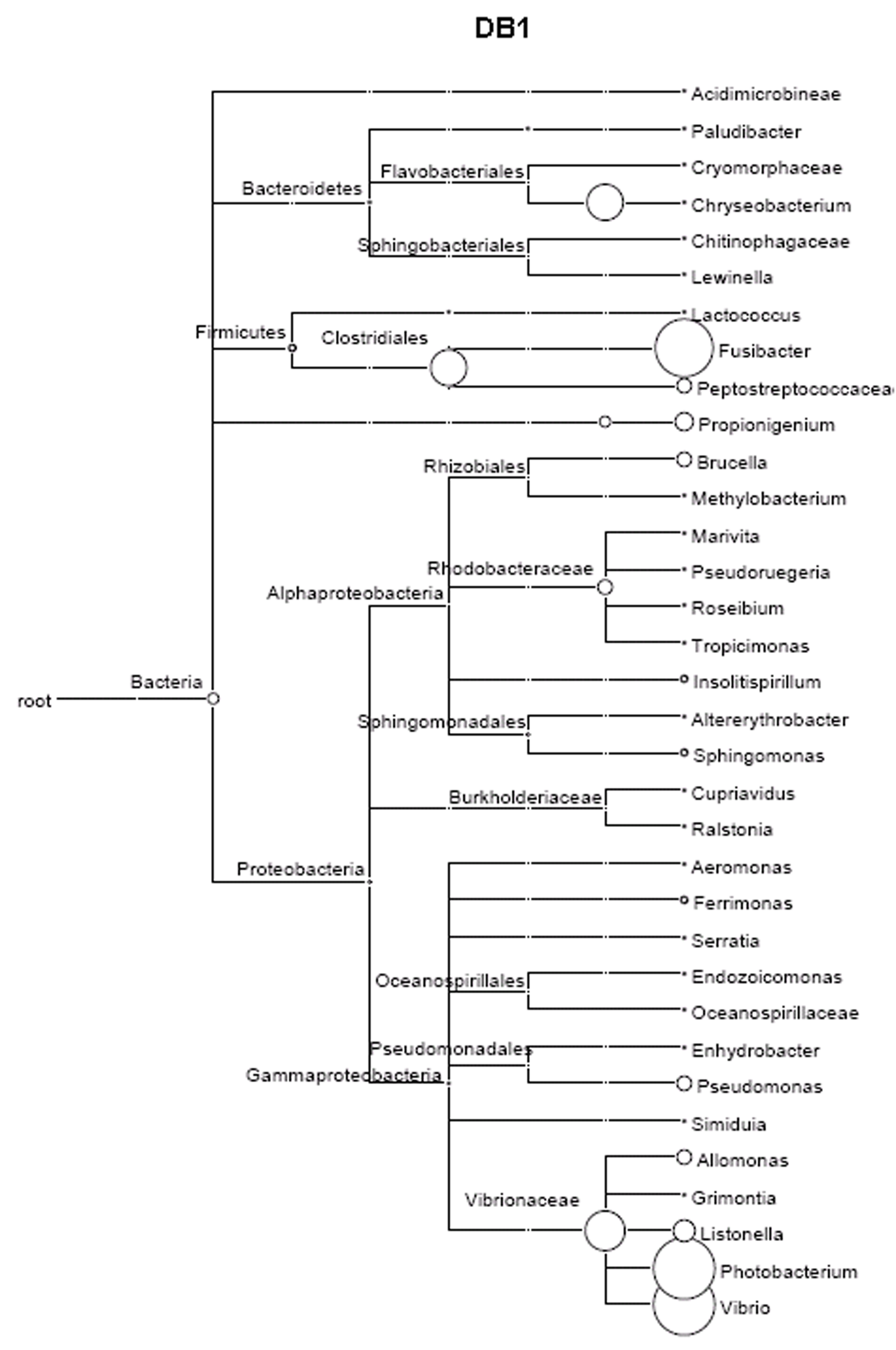

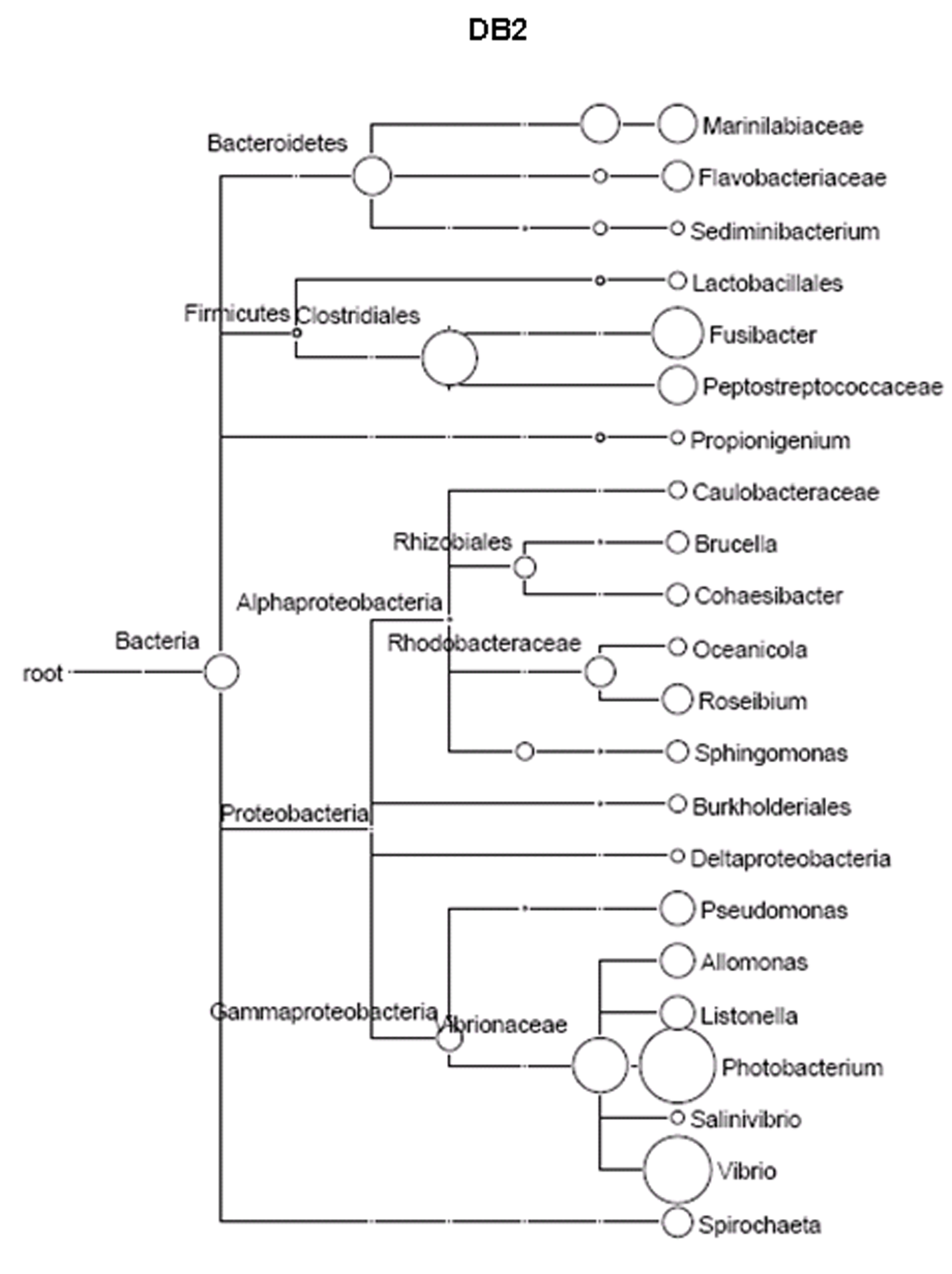

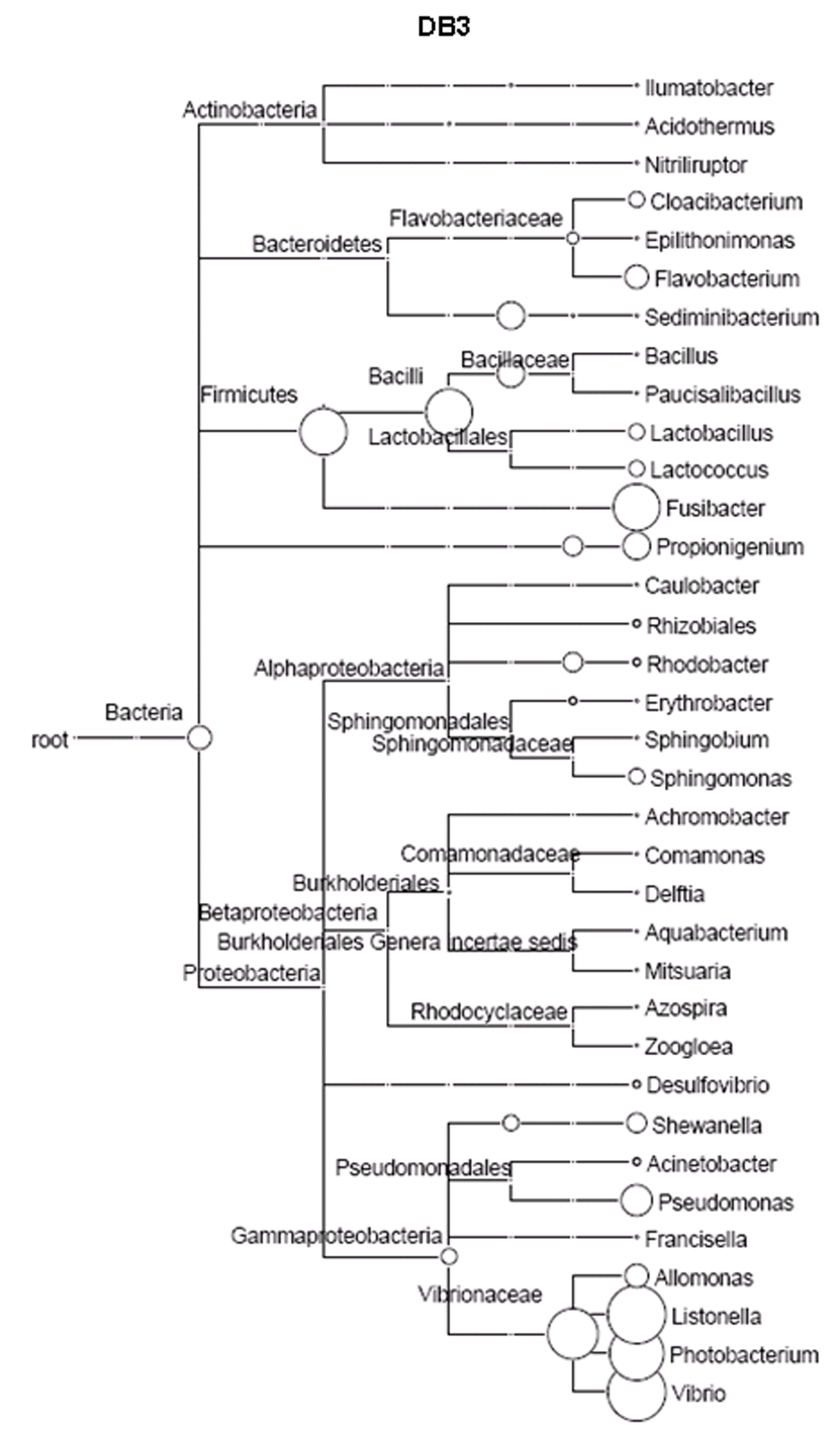
**

Supplement: Figure S1 — The hierarchical trees reflect bacterial abundance for (A) wild-caught shrimp (WC1, WC2, and WC3) and (B) domesticated shrimp (DB1, DB2, and DB3). (DOC) [file pone.0091853.s001.doc]
