# Supplementary material for: Characterization of Intestinal Bacteria in Wild and Domesticated Adult Black Tiger Shrimp (Penaeus monodon)
Source: PLoS One. 2014 Mar 11;9(3):e91853. doi: 10.1371/journal.pone.0091853 (PMC3950284; doi:10.1371/journal.pone.0091853)
Supplement: Table S1 — Taxonomical assignments of 16S rRNA sequences from pyrosequencing using the RDP classifier with a confidence threshold of 80%. (DOC) [file pone.0091853.s002.doc]

**Table S1.** Taxonomical assignments of 16S rRNA sequences from pyrosequencing using the RDP classifier with a confidence threshold of 80%

| **Phylum** | **Classification** | **WC** |  |  | **DB** |  |  |
| --- | --- | --- | --- | --- | --- | --- | --- |
|  |  | **1** | **2** | **3** | **1** | **2** | **3** |
| ***Actinobacteria*** |  |  |  |  |  |  |  |
|  | *Brevibacterium* | 1 | 5 |  |  |  |  |
|  | *Corynebacterium* |  | 1 |  |  | 1 |  |
|  | *Ilumatobacter* |  | 3 |  |  |  | 1 |
|  | *Kocuria* | 1 |  |  |  |  |  |
|  | *Nitriliruptor* |  |  |  |  |  | 1 |
|  | *Patulibacter* |  |  |  |  | 2 |  |
|  | *Renibacterium* |  | 8 |  |  |  |  |
|  | Unclassified *Micrococcaceae* |  | 4 |  |  |  |  |
|  | Unclassified *Acidimicrobineae* |  | 1 | 1 | 1 |  |  |
|  | Unclassified *Frankineae* |  | 1 |  |  |  |  |
|  | Unclassified *Micrococcineae* |  |  | 1 |  |  |  |
|  | Unclassified *Actinomycetales* |  | 1 | 1 |  |  | 2 |
|  | Unclassified *Actinobacteria* |  | 1 |  | 1 | 1 | 1 |
|  | **Total** | 2 | 25 | 3 | 2 | 4 | 5 |
|  |  |  |  |  |  |  |  |
| ***Bacteroidetes*** |  |  |  |  |  |  |  |
|  | *Aequorivita* |  | 1 |  |  |  |  |
|  | *Algoriphagus* |  | 20 |  |  |  |  |
|  | *Aquimarina* |  |  | 11 |  |  |  |
|  | *Chryseobacterium* |  | 3 |  | 1 | 1 |  |
|  | *Cloacibacterium* | 1 | 10 | 3 |  |  | 4 |
|  | *Crocinitomix* | 1 |  | 2 |  | 1 |  |
|  | *Delftia* |  |  |  |  |  | 1 |
|  | *Epilithonimonas* |  | 1 |  |  |  | 1 |
|  | *Fabibacter* |  |  | 5 |  |  |  |
|  | *Flavobacterium* |  | 26 | 2 |  |  | 5 |
|  | *Francisella* |  |  |  |  |  | 1 |
|  | *Gilvibacter* | 7 | 4 |  |  |  |  |
|  | *Haliscomenobacter* |  | 1 |  |  |  |  |
|  | *Lewinella* |  |  | 4 | 1 |  |  |
|  | *Lishizhenia* | 2 | 25 |  |  |  |  |
|  | *Marinifilum* |  |  | 1 |  |  |  |
|  | *Mesoflavibacter* |  | 3 | 2 |  | 1 |  |
|  | *Pedobacter* |  | 5 | 2 |  | 1 |  |
|  | *Porphyromonas* |  |  |  |  | 1 |  |
|  | *Psychroserpens* |  |  | 2 |  |  |  |
|  | *Roseateles* |  |  |  |  |  | 1 |
|  | *Salisaeta* |  |  | 1 |  |  |  |
|  | *Sediminibacterium* |  | 1 |  |  |  |  |
|  | *Sphingobacterium* |  |  | 1 |  |  |  |
|  | *Tenacibaculum* | 2 | 26 | 2 |  |  |  |
|  | Unclassified *Chitinophagaceae* |  | 13 |  |  | 9 | 1 |
|  | Unclassified *Cryomorphaceae* | 1 | 1 | 25 | 1 |  |  |
|  | Unclassified *Cytophagaceae* |  | 1 |  | 1 | 1 |  |
| **Phylum** | **Classification** | **WC** |  |  | **DB** |  |  |
|  |  | **1** | **2** | **3** | **1** | **2** | **3** |
|  | Unclassified *Flavobacteriaceae* |  | 42 | 48 | 57 | 20 | 6 |
|  | Unclassified *Saprospiraceae* |  |  | 18 |  |  |  |
|  | Unclassified *Sphingobacteriaceae* |  | 6 |  |  |  |  |
|  | Unclassified *Bacteroidales* |  |  | 1 |  | 1 |  |
|  | Unclassified *Flavobacteriales* | 2 | 13 | 7 | 8 | 4 |  |
|  | Unclassified *Sphingobacteriales* |  | 69 | 6 | 2 |  | 5 |
|  | Unclassified *Bacteroidetes* |  | 123 | 54 | 3 | 122 | 11 |
|  | **Total** | 16 | 394 | 197 | 74 | 162 | 36 |
|  |  |  |  |  |  |  |  |
| ***Firmicutes*** |  |  |  |  |  |  |  |
|  | *Bacillus* |  |  |  |  |  | 1 |
|  | *Clostridium XI* |  |  |  | 5 |  |  |
|  | *Exiguobacterium* |  | 190 |  |  |  |  |
|  | *Fusibacter* | 2 | 8 | 76 | 655 | 130 | 64 |
|  | *Gemella* |  | 2 |  |  |  |  |
|  | *Lactobacillus* | 4 | 6,454 | 3 |  | 4 | 4 |
|  | *Lactococcus* | 1 | 10 | 2 | 1 | 1 | 4 |
|  | *Macrococcus* |  | 2 |  |  | 1 |  |
|  | *Paenibacillus* |  |  | 1 |  |  |  |
|  | *Pediococcus* |  | 33 |  |  |  |  |
|  | *Staphylococcus* |  | 8 |  |  |  |  |
|  | *Streptococcus* | 1 | 3 |  |  | 1 |  |
|  | *Weissella* | 2 |  |  |  |  |  |
|  | Unclassified *Lactobacillaceae* |  | 110 |  |  |  |  |
|  | Unclassified *Peptostreptococcaceae* | 1 |  | 9 | 31 | 22 |  |
|  | Unclassified *Clostridiales* |  | 1 | 67 | 53 | 220 |  |
|  | Unclassified *Clostridiales Incertae Sedis XII* |  |  | 1 | 5 |  |  |
|  | Unclassified *Bacillales* |  | 1 |  |  | 1 | 1 |
|  | Unclassified *Bacilli* |  | 5 |  | 1 |  |  |
|  | Unclassified *Clostridia* |  |  |  | 1 |  |  |
|  | Unclassified *Firmicutes* | 28 | 9 | 326 | 2 | 3 | 72 |
|  | **Total** | 39 | 6,836 | 485 | 754 | 383 | 146 |
|  |  |  |  |  |  |  |  |
| ***Fusobacteria*** |  |  |  |  |  |  |  |
|  | *Propionigenium* | 1 |  |  | 1 |  |  |
|  | Unclassified *Fusobacteriaceae* | 2 | 3 | 1 | 13 | 8 | 18 |
|  | Unclassified *Lactobacillales* |  | 55 |  |  |  |  |
|  | **Total** | 3 | 58 | 1 | 14 | 8 | 18 |
|  |  |  |  |  |  |  |  |
| ***Proteobacteria*** |  |  |  |  |  |  |  |
| ***a-Proteobacteria*** | *Altererythrobacter* |  |  | 2 |  |  |  |
|  | *Bosea* |  | 1 |  |  |  |  |
|  | *Bradyrhizobium* |  | 6 |  |  | 2 |  |
|  | *Brevundimonas* | 3 | 40 |  |  | 3 |  |
|  | *Brucella* | 1 | 19 | 1 | 5 | 8 |  |
|  | *Caulobacter* |  | 12 |  |  | 3 | 1 |
| **Phylum** | **Classification** | **WC** |  |  | **DB** |  |  |
|  |  | **1** | **2** | **3** | **1** | **2** | **3** |
|  | *Cohaesibacter* | 4 | 5 |  |  | 10 |  |
|  | *Devosia* |  |  |  |  | 1 |  |
|  | *Donghicola* |  | 3 |  |  |  |  |
|  | *Erythrobacter* |  | 3 |  |  | 1 |  |
|  | *Filomicrobium* |  | 8 |  |  |  |  |
|  | *Hyphomonas* |  | 1 |  |  |  |  |
|  | *Jannaschia* |  |  | 1 |  |  |  |
|  | *Kiloniella* |  |  | 2 |  |  |  |
|  | *Labrenzia* |  | 12 |  |  | 3 |  |
|  | *Loktanella* |  | 3 | 1 |  |  |  |
|  | *Methylobacterium* | 1 | 19 | 4 | 1 | 1 |  |
|  | *Nautella* | 2 | 3 |  |  |  |  |
|  | *Nitratireductor* |  | 1 |  |  |  |  |
|  | *Novosphingobium* |  | 15 |  |  |  |  |
|  | *Oceanicola* | 1 | 5 |  |  | 1 |  |
|  | *Paracoccus* |  | 17 |  |  |  |  |
|  | *Phaeobacter* | 2 | 2 |  |  | 1 |  |
|  | *Phenylobacterium* |  | 16 | 1 |  | 1 |  |
|  | *Pseudoruegeria* |  | 7 |  |  |  |  |
|  | *Rhodobacter* |  | 2 |  |  |  | 2 |
|  | *Roseibium* |  | 1 | 1 |  | 1 |  |
|  | *Roseovarius* | 1 | 15 | 2 |  |  |  |
|  | *Silicibacter* |  | 1 |  | 1 |  |  |
|  | *Shimia* |  | 20 | 7 |  |  |  |
|  | *Sphingobium* |  | 11 | 1 |  |  | 1 |
|  | *Sphingomonas* | 4 | 47 | 12 | 3 | 9 | 4 |
|  | *Thalassobius* | 5 | 25 |  |  | 1 |  |
|  | *Xanthobacter* |  |  | 3 |  |  |  |
|  | Unclassified *Brucellaceae* |  |  |  |  | 1 |  |
|  | Unclassified *Hyphomicrobiaceae* |  | 1 | 1 |  | 1 |  |
|  | Unclassified *Phyllobacteriaceae* |  | 3 |  |  | 2 |  |
|  | Unclassified *Rhodobacteraceae* | 86 | 515 | 31 | 9 | 45 | 5 |
|  | Unclassified *Rhodospirillaceae* |  |  | 1 | 3 |  |  |
|  | Unclassified *Sphingomonadaceae* |  | 22 | 5 |  | 2 |  |
|  | Unclassified *Rhizobiales* |  | 2 |  |  | 1 | 1 |
|  | Unclassified *Sphingomonadales* | 4 | 38 | 6 | 3 | 6 | 4 |
|  | Unclassified *Alphaproteobacteria* |  |  |  | 1 | 2 | 2 |
|  | **Total** | 114 | 901 | 82 | 26 | 106 | 20 |
|  |  |  |  |  |  |  |  |
| ***β-Proteobacteria*** | *Achromobacter* |  | 1 |  |  |  | 1 |
|  | *Acidovorax* |  | 12 | 4 |  |  |  |
|  | *Aquabacterium* |  | 12 |  |  |  | 1 |
|  | *Azonexus* |  | 1 |  |  |  |  |
|  | *Azospira* |  | 11 | 2 |  |  | 1 |
|  | *Bordetella* |  | 2 |  |  |  |  |
|  | *Comamonas* |  | 3 |  |  | 1 | 1 |
| **Phylum** | **Classification** | **WC** |  |  | **DB** |  |  |
|  |  | **1** | **2** | **3** | **1** | **2** | **3** |
|  | *Cupriavidus* |  | 3 |  | 1 |  |  |
|  | *Curvibacter* |  | 1 |  |  | 1 |  |
|  | *Herbaspirillum* |  | 1 |  |  | 1 |  |
|  | *Janthinobacterium* | 2 | 2 | 4 |  |  |  |
|  | *Maritalea* |  |  | 1 |  |  |  |
|  | *Mesorhizobium* |  |  | 1 |  |  |  |
|  | *Methylotenera* | 1 |  |  |  |  |  |
|  | *Methyloversatilis* |  | 6 |  |  |  |  |
|  | *Pelomonas* |  | 3 |  |  | 1 |  |
|  | *Polynucleobacter* |  | 1 |  |  |  |  |
|  | *Ralstonia* | 1 |  | 1 | 1 |  |  |
|  | *Sandaracinobacter* |  |  | 1 |  |  |  |
|  | *Variovorax* |  | 3 |  |  |  |  |
|  | *Zoogloea* |  |  | 2 |  |  | 1 |
|  | *Undibacterium* | 1 | 9 | 2 |  | 3 |  |
|  | Unclassified *Alcaligenaceae* |  | 1 |  |  |  |  |
|  | Unclassified *Comamonadaceae* | 1 | 3 |  |  |  |  |
|  | Unclassified *Neisseriaceae* |  | 1 | 1 |  |  |  |
|  | Unclassified *Burkholderiales* |  | 1 | 1 |  |  | 1 |
|  | Unclassified *Betaproteobacteria* |  |  | 4 |  | 1 |  |
|  | **Total** | 6 | 77 | 24 | 2 | 8 | 6 |
|  |  |  |  |  |  |  |  |
| ***d-Proteobacteria*** |  |  |  |  |  |  |  |
|  | *Desulfovibrio* |  |  | 61 |  | 1 | 2 |
|  | Unclassified *Bacteriovoracaceae* |  |  | 1 |  |  |  |
|  | Unclassified *Desulfobulbaceae* |  |  |  |  | 1 |  |
|  | Unclassified *Desulfovibrionaceae* |  |  |  |  | 1 |  |
|  | Unclassified *Oxalobacteraceae* |  |  | 1 |  |  |  |
|  | Unclassified *Nannocystineae* |  |  |  |  | 1 |  |
|  | Unclassified *Desulfovibrionales* |  | 1 |  |  |  |  |
|  | Unclassified *Myxococcales* |  |  | 1 |  |  |  |
|  | Unclassified *Deltaproteobacteria* |  |  | 2 |  |  |  |
|  | **Total** |  | 1 | 66 |  | 4 | 2 |
| ***e-Proteobacteria*** |  |  |  |  |  |  |  |
|  | *Arcobacter* |  |  | 52 |  |  |  |
|  | Unclassified *Campylobacteraceae* |  |  | 2 |  |  |  |
|  | **Total** |  |  | 54 |  |  |  |
|  |  |  |  |  |  |  |  |
| ***g-Proteobacteria*** |  |  |  |  |  |  |  |
|  | *Acinetobacter* |  | 41 | 9 |  | 2 | 2 |
|  | *Aeromonas* |  |  |  | 1 |  |  |
|  | *Agarivorans* |  |  | 4 |  |  |  |
|  | *Alcanivorax* |  | 2 |  |  |  |  |
|  | *Allomonas* | 6 | 1 |  | 1 | 4 | 1 |
|  | *Alteromonas* | 4 |  |  |  |  |  |
| **Phylum** | **Classification** | **WC** |  |  | **DB** |  |  |
|  |  | **1** | **2** | **3** | **1** | **2** | **3** |
|  | *Amphritea* |  | 5 |  |  |  |  |
|  | *Endozoicomonas* |  |  |  | 1 | 2 |  |
|  | *Enhydrobacter* | 1 | 12 |  | 1 |  |  |
|  | *Escherichia/Shigella* | 4 | 6 | 1 |  | 1 |  |
|  | *Ferrimonas* |  | 56 |  | 3 |  |  |
|  | *Grimontia* |  |  |  |  | 1 |  |
|  | *Haemophilus* |  | 3 |  |  |  |  |
|  | *Halomonas* | 1 |  |  |  |  |  |
|  | *Legionella* |  |  | 1 |  | 1 |  |
|  | *Listonella* | 1 | 2 |  |  |  | 3 |
|  | *Marinicella* |  | 1 |  |  |  |  |
|  | *Marinomonas* |  |  | 1 |  |  |  |
|  | *Methylophaga* |  | 8 |  |  |  |  |
|  | *Neptuniibacter* | 3 |  |  |  |  |  |
|  | *Oceanospirillum* | 1 |  |  |  |  |  |
|  | *Photobacterium* | 1,710 | 297 | 5,978 | 901 | 1,218 | 102 |
|  | *Pseudoalteromonas* |  | 3 | 1 |  |  |  |
|  | *Pseudomonas* | 17 | 402 | 22 | 5 | 32 | 16 |
|  | *Serratia* |  | 2 |  |  |  |  |
|  | *Shewanella* |  | 1 | 1 |  |  |  |
|  | *Stenotrophomonas* | 1 | 1 |  |  |  |  |
|  | *Thalassomonas* |  |  | 2 |  |  |  |
|  | *Vibrio* | 1,894 | 250 | 1,009 | 847 | 422 | 38 |
|  | Unclassified *Alteromonadaceae* | 1 |  |  |  |  |  |
|  | Unclassified *Enterobacteriaceae* |  | 9 |  | 1 | 2 |  |
|  | Unclassified *Moraxellaceae* |  |  | 1 |  |  |  |
|  | Unclassified *Oceanospirillales* |  |  | 1 |  |  |  |
|  | Unclassified *Pasteurellaceae* |  |  |  |  | 1 |  |
|  | Unclassified *Pseudomonadaceae* |  | 73 | 2 |  |  |  |
|  | Unclassified *Vibrionaceae* | 1,150 | 424 | 2,685 | 302 | 738 | 500 |
|  | Unclassified *Alteromonadales* |  |  | 2 |  |  | 3 |
|  | Unclassified *Gammaproteobacteria* | 21 | 41 | 332 | 16 | 40 | 17 |
|  | **Total** | 4,815 | 1,640 | 10,052 | 2,079 | 2,464 | 682 |
|  |  |  |  |  |  |  |  |
| **Unclassified *Proteobacteria*** | Unclassified *Proteobacteria* | 1 | 2 | 2 | 2 | 2 |  |
|  | **Total** | 1 | 2 | 2 | 2 | 2 |  |
|  |  |  |  |  |  |  |  |
| **Unclassified Bacteria** | Unclassified Bacteria | 33 | 35 | 566 | 8 | 49 | 94 |
|  | **Total** | 33 | 35 | 566 | 8 | 49 | 94 |
| **Total sequences** |  | 5,029 | 9,969 | 11,532 | 2,961 | 3,190 | 1,009 |
